# Divergent responses to urbanisation and forest cover shape the spatial distribution of *Culex pipiens* ecotypes in western Switzerland

**DOI:** 10.1186/s13071-026-07533-8

**Published:** 2026-06-26

**Authors:** Antoine Perrin, Clara Deillon, Philippe Christe, Olivier Glaizot

**Affiliations:** 1 Department of Ecology and Evolution, University of Lausanne, UNIL-Sorge, Biophore, 1015 Lausanne, Switzerland; 2 Department of Zoology, State Museum of Natural Sciences, 1014 Lausanne, Switzerland

**Keywords:** *C. p. pipiens*, *C. p. molestus*, Hybrids, Bioform, Seasonal variation, Agricultural, Scale-of-effect

## Abstract

**Background:**

Arthropod-borne viruses are increasingly reported across Europe, with *Culex pipiens* serving as the main vector of West Nile and Usutu viruses. The *C. pipiens* complex comprises two ecologically distinct ecotypes, *pipiens* and *molestus*, which can hybridise. These ecotypes are thought to differ in habitat preference, with *C. p. molestus* more frequent in urban environments and *C. p. pipiens* in natural ones. However, many studies have either not distinguished between these ecotypes or have investigated them without considering a clear landscape gradient. This information is nevertheless important for understanding vector distribution and the potential implications for virus transmission pathways and spillover risk.

**Methods:**

A season-long survey was conducted from May to September 2025 across 57 sampling sites in Switzerland. Mosquitoes were morphologically identified, and *C. pipiens* ecotypes were distinguished using molecular analyses. We characterised landscape composition and their scale of effects, as well as meteorological conditions on the sampling dates to determine their influence on the presence and relative proportion of each *C. pipiens* ecotype.

**Results:**

Agricultural landscapes promoted all ecotypes, urbanisation positively influenced *C. p. pipiens* but not *C. p. molestus*, and forest cover reduced the presence and relative proportion of hybrids. In addition, temperature was positively correlated with all ecotype presence, whereas rainfall had no detectable effect.

**Conclusions:**

The responses to landscape variables differ among *C. pipiens* ecotypes and across different landscape features. This highlights the complex, context-dependent interactions between the ecology of mosquitoes and the composition of their habitat, which ultimately determine their spatial distribution patterns.

**Graphical Abstract:**

**Figure:**
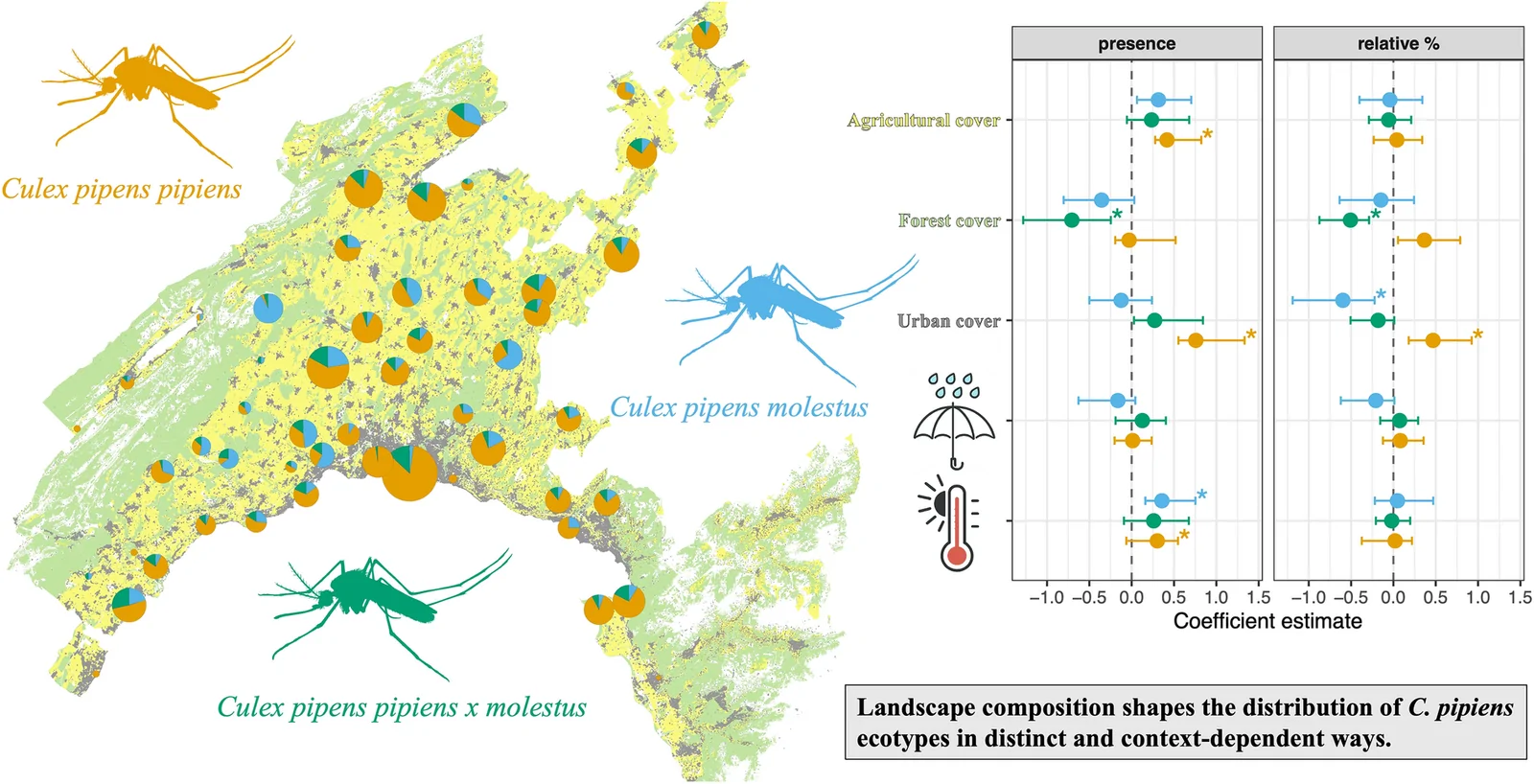

**Supplementary Information:**

The online version contains supplementary material available at https://doi.org/10.1186/s13071-026-07533-8.

## Background

Arthropod-borne viruses of medical and veterinary importance are increasingly reported across Europe, with recurrent transmission documented in multiple regions, including areas that had not previously recorded infections [1–3]. This spread is largely linked to environmental changes, especially rising temperatures and habitat fragmentation, which promote the transmission and persistence of arboviruses [4, 5]. Such changes act through shifts in the distribution, abundance, and seasonal dynamics of mosquito vectors such as *Culex pipiens*, the main European vector of West Nile and Usutu viruses [6–8], increasing opportunities for virus transmission.

The *C. pipiens* complex is ecologically heterogeneous and includes two ecotypes with distinct biological and behavioural traits [9]. The *pipiens* form is typically associated with aboveground habitats, requires a bloodmeal to lay eggs, overwinters in diapause and is primarily ornithophilic, whereas the *molestus* form is mostly found in subterranean environments, can oviposit once without a bloodmeal, remains active during winter and exhibits a higher degree of mammal-feeding behaviour [10, 11]. Hybrids between these two ecotypes display intermediate ecological characteristics and are frequently highlighted as potential epidemiological bridge vectors [12, 13]. As a result, the relative distribution and co-occurrence of these ecotypes may have important ecological and epidemiological implications.

Given their contrasting ecological characteristics, a higher frequency of *C. p. molestus* is expected in urban environments, whereas *C. p. pipiens* is assumed to be more frequent in natural or rural habitats [10, 14–16]. However, many studies have either not distinguished between the two ecotypes or have investigated them without considering a clear landscape gradient. The few studies explicitly addressing this issue report mixed results. Some support the expected urban-natural segregation pattern [16–18], and others report the opposite trend [19, 20]. Indeed, the distribution of the two ecotypes appears highly context dependent. In northern Europe, *C. p. pipiens* and *C. p. molestus* tend to show an allopatric distribution with limited hybridisation, whereas they more frequently occur in sympatry in southern regions, with substantial levels of hybridisation [12, 15, 21]. Together, these findings suggest that broad generalisations are difficult and that the distribution and ecological segregation of the two ecotypes should be assessed at the regional or local scale.

To understand the factors influencing the distribution of *C. pipiens* ecotypes, it is necessary to move beyond dichotomic urban-rural contrasts and explicitly distinguish the main components of landscape anthropisation, such as urban areas, agricultural land and forest cover. Although these components are often spatially correlated and causally interconnected [22–25], they can exert distinct ecological pressures through the loss and fragmentation of favourable micro-habitats, altered resource availability, or shifts in host community composition for mosquitoes. Disentangling the relative contribution of urban, agricultural and forest cover is essential for developing targeted management or conservation strategies adapted to specific landscape contexts [26, 27]. Importantly, the influence of a landscape structure on a given ecological process, such as mosquito population dynamics, is inherently scale-dependent [28]. The spatial extent at which environmental variables affect mosquito presence, abundance, or hybrid occurrence may vary according to their ecological traits and dispersal capacity [29–31]. Moreover, even for a given species-landscape combination, the scale of effect can differ depending on the response variable considered, highlighting the need to determine it empirically rather than assuming it a priori [32].

This study involved a season-long survey of mosquito populations across the canton of Vaud in Switzerland, a region characterised by a mosaic of urban (10%), agricultural (42%), and forested (32%) areas, along with 16% of other unproductive surfaces, mainly consisting of water bodies and mountainous areas with sparse vegetation (e.g. rocks, scree, glaciers) [33]. A total of 57 sampling sites were selected to capture variation in urban, agricultural, and forested environments. The objectives were to (i) describe the spatio-temporal distribution of *C. pipiens* ecotypes (*pipiens*, *molestus*, and hybrids); and (ii) identify the main environmental factors associated with their presence and relative proportions, with a particular focus on landscape composition and meteorological conditions. Based on their known ecological traits, we hypothesised that the seasonal dynamics of *C. p. molestus* would be more stable than *C. p. pipiens* due to its year-round activity without diapause. Additionally, we predicted that *C. p. molestus* would be more prevalent in urbanised environments due to its adaptation to confined habitats and preference for mammalian hosts, whereas *C. p. pipiens* would be more frequent in natural or rural areas, where open habitats and higher densities of avian hosts are found [9]. Furthermore, we expected *C. p. molestus* to exhibit greater temperature sensitivity, reflecting its relatively low presence above ground in northern Europe and its widespread activity in southern regions [9, 21]. Finally, we expected hybrids to display intermediate spatial and temporal patterns between the two ecotypes. By providing detailed information on the distribution and ecology of these *C. pipiens* ecotypes across different landscape contexts, this study aims to inform local vector surveillance and improve our understanding of the ecological factors shaping mosquito populations.

## Methods

### Mosquito sampling

**Fig. 1:**
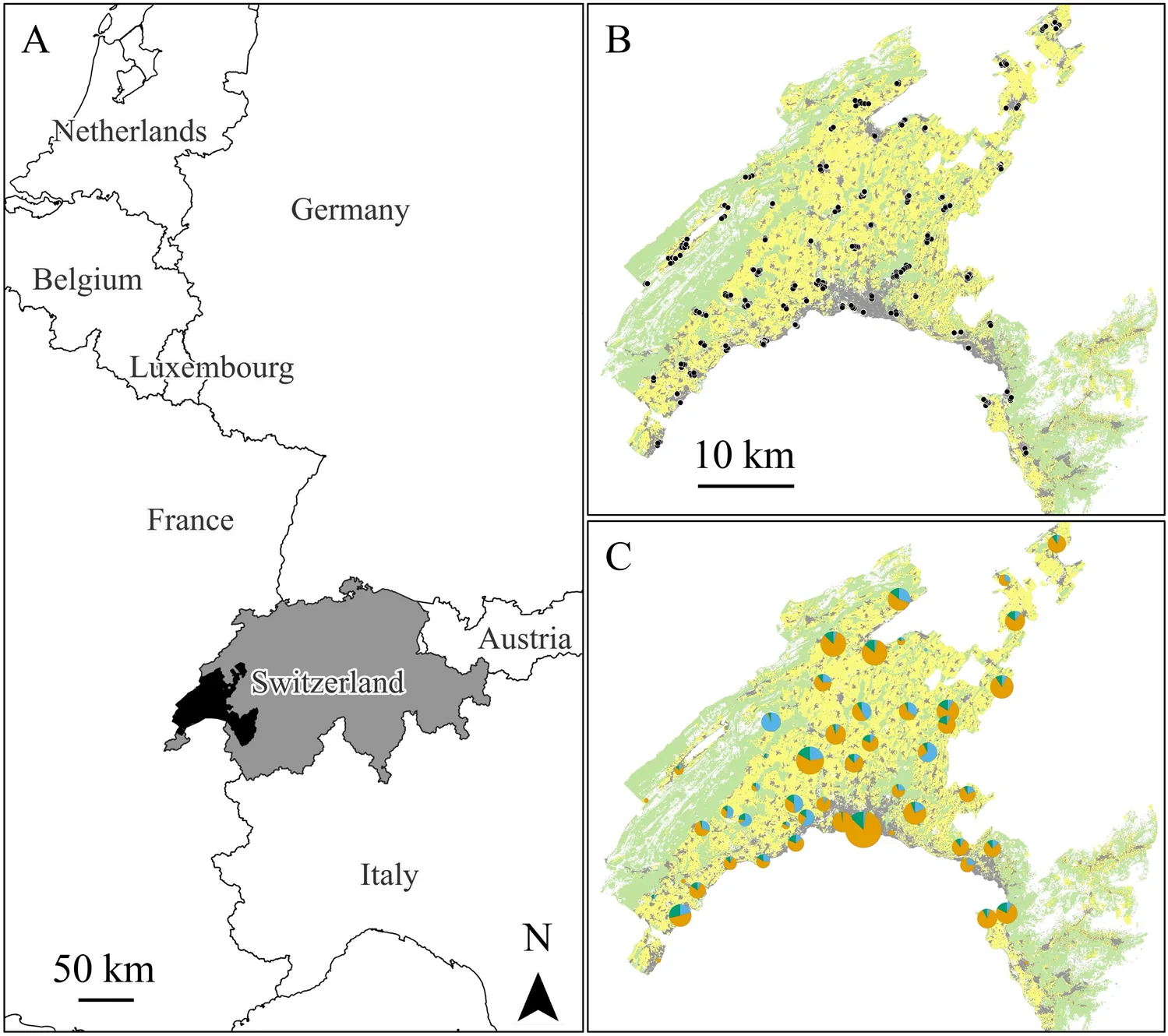
Study area and distribution of *C. pipiens* ecotypes in the canton of Vaud, Switzerland. **A** Location of the study area within Europe; Switzerland is highlighted in grey and the canton of Vaud in black. **B** Distribution of mosquito traps (black points) across the canton. **C** Relative composition of *C. pipiens* ecotypes at sampling sites where at least one individual was captured. Pie charts represent the proportion of form *pipiens* (orange), hybrids (green), and form *molestus* (blue). Light yellow, light green, and grey background colours in panels B and C represent agricultural (i.e. Landw. Bewirtschaftung: Nutzungsflächen shapefile-geodienste.ch), forest (i.e. entities ‘Gebueschwald’, ‘Wald’, ‘Wald offen’, and ‘Gehoelzflaeche’ from the Bodenbedeckung shapefile-swissTLM3D-swisstopo) and urban (i.e. Gebaeude_Footprint shapefile-swissTLM3D-swisstopo) areas, respectively

Mosquito captures were conducted from May 8 to September 17, 2025, using CO₂-baited BG-Pro traps (Biogents AG, Regensburg, Germany) in CDC style configuration. CO₂ was supplied from compressed gas cylinders (Carbagas, Switzerland) and released at an approximate rate of 0.5 kg/24 h during trapping, as recommended by the trap manufacturer. During the study period, 56 sampling sessions were conducted across 57 sites distributed throughout the canton of Vaud (Fig. 1A). Each sampling session consisted of simultaneous trapping at three sites, with five traps per site (i.e. 15 traps used per session), set at approximately 5 pm and retrieved at around 9 am the following morning. Within each site, traps were spaced with a mean distance of 469 m (Fig. 1B). Sites were visited between one and ten times depending on the number of *C. pipiens* collected during the first sessions, and a site was considered sufficiently sampled once at least 20 *C. pipiens* specimens had been captured. This sampling design prioritised spatial coverage across the study area over intensive sampling at individual sites. Captured mosquitoes were morphologically identified using the key of Becker et al. [10]. As adult females of *Culex torrentium* are morphologically indistinguishable from *C. pipiens*, individuals belonging to *C. pipiens/torrentium* were separated from other species and individually stored in sterile 1.5 mL tubes containing 70% ethanol. Only a few males were collected and were therefore not identified to species level. Samples were subsequently preserved at −20 °C until molecular analyses were performed.

### Molecular identification of mosquitoes

DNA was extracted from the legs and wings of each mosquito. To prevent cross-contamination, each specimen was dissected individually on a clean disposable surface. Dissection tools were disinfected with 100% ethanol and flame-sterilised between samples. For each sample, 20 µL of proteinase K (Promega, Madison, WI, USA; 600 U/mL) was added prior to mechanical homogenisation using sterile pestles. Subsequently, 180 µL of ATL buffer was added, and samples were centrifuged and incubated at 56 °C for 2 h 30 min. DNA extraction was performed using the BioSprint 96 DNA Blood Kit (Qiagen, Hilden, Germany) on a BioSprint 96 Workstation (Qiagen), following the manufacturer’s protocol for ‘Purification of DNA from Tissues’, with minor modifications. DNA was eluted in 100 µL of Milli-Q water instead of 200 µL of AE buffer, and 500 µL of Buffer AW1 was used per well during the “Load Wash 1” step instead of 650 µL.

Species (*C. pipiens*/*torrentium*) and ecotype (*C. p. pipiens*/*molestus* and hybrids) identifications were performed using two different PCR assays targeting the ACE locus [34] and the CQ11 microsatellite marker [35], respectively. Both reactions were carried out in a final volume of 20 µL containing 4 µL of 5X buffer, 2 mM MgCl_2_, 0.2 mM dNTPs, 0.025 U/µL Taq DNA polymerase, 2 µL of template DNA, and nuclease-free water to volume. Primer combinations and final concentrations were as follows: for ACE, 0.15 µM ACEpip-F, 0.1 µM ACEtorr-F, and 0.15 µM B1246s-R; for CQ11, 0.15 µM CQ11F2, 0.1 µM pipCQ11R, and 0.15 µM molCQ11R.

PCR products were separated by electrophoresis on 2% agarose gels and DNA fragments were visualised under UV illumination to determine species identity (*C. pipiens* or *C. torrentium*) and, for *C. pipiens*, the corresponding ecotype (*pipiens*, *molestus*, or hybrid). Gel electrophoresis results were independently interpreted by two trained persons, with discrepancies resolved through consensus.

### Meteorological data extraction and interpolation

Daily meteorological data were obtained from MeteoSwiss automatic and manual weather stations through the Federal Geoportal (data.geo.admin.ch). For each station, daily mean air temperature (tre200d0; 2 m above ground) and daily sum of rainfall (rre150d0) were downloaded for the sampling period. Meteorological stations located within a 30 km radius of at least one mosquito trap were included to ensure local representativeness of weather conditions. Elevation for both meteorological stations and mosquito trap locations was extracted from the SwissALTI3D digital elevation model.

To estimate meteorological conditions at each mosquito trap location, spatial interpolation was performed using Regression Kriging. For each day and each meteorological variable (temperature and rainfall), a regression model including spatial coordinates and altitude as predictors was fitted to station data, and kriging of residuals was applied to generate predicted values at trap locations. The best-fitting variogram model was automatically selected within the autoKrige function. Negative interpolated rainfall values were set to zero. For each trap, meteorological variables were then matched to the sampling date to obtain the daily temperature and rainfall over the 24-h period spanning the day before and the night of sampling, from 6 am to 6 am the next morning. In addition, mean temperature and cumulative rainfall over the seven and 14 days preceding each sampling event were calculated to account for potential delayed effects on mosquito dynamics. As results were qualitatively similar across temporal aggregations, only the 24-h variables are presented in the main text, while analyses based on 7- and 14-day averages are provided in the Supplementary Materials (Fig. S1). All analyses were conducted in R (version 4.5.2). Spatial data processing was performed using the packages *sf* and *terra*, and Regression Kriging was implemented using the *automap* package.

### Landscape characterisation

Landscape composition around each mosquito trap was quantified following the same spatial data sources and analytical framework described in Perrin et al. [36]. Briefly, we used the swissTLM3D geodata provided by the Federal Office of Topography Swisstopo to derive urban and forest cover from the *Gebaeude_Footprint* and *Bodenbedeckung* layers (including the entities *Gebueschwald*, *Wald*, *Wald offen*, and *Gehoelzflaeche*), respectively. Agricultural areas were defined using the *Landw. Bewirtschaftung: Nutzungsflächen* shapefile (version 1.4), available from Swiss cantonal geodata services (https://www.geodienste.ch/). For each trap, the proportion of forest, urban, and agricultural areas was calculated within concentric circular buffers. Buffer radii ranged from 25 to 2500 m at 25 m intervals, resulting in 100 spatial scales per site. The smallest buffer represented the immediate surroundings of the trap location, whereas the largest buffer encompassed a landscape extent well beyond the mean flight distance reported for *C. pipiens* (402–475 m [37]), following standard scale-of-effect approaches [28, 38]. Two sampling sites located close to the Swiss border were excluded from the landscape analyses. Because of their proximity to the national boundary, landscape data could not be extracted at the full spatial extent required for the scale-of-effect analysis. In addition, very few mosquitoes were collected at these two sampling sites.

### Scale-of-effect analysis

For each trap, we recorded the presence or absence of each *C. pipiens* ecotype. For traps containing at least one *C. pipiens* individual, we additionally quantified the relative frequency of each ecotype (*pipiens*, *molestus*, or hybrid). The presence–absence models capture variation in the probability of occurrence of each *C. pipiens* ecotype across the landscape, whereas the relative proportion models describe changes in the composition of co-occurring ecotypes within sites where *C. pipiens* is present. Together, these complementary approaches allow disentangling changes in distribution from changes in community composition.

We assessed the scale of effect of each landscape component (urban, forest, and agricultural cover) on *C. pipiens* ecotype presence and relative frequency independently across the 100 spatial scales. For each response variable, we fitted a series of generalised additive models (GAMs) using the R package *mgcv*, spanning the full range of buffer sizes (25–2500 m). Each model included a single landscape component at a given spatial scale, while accounting for site-level and seasonal structure. Sampling site was included as a random-effect term, and capture date was modelled as a smooth temporal function. Binomial error distributions were used in all models. For relative frequency models, the binomial response (cbind(pipiens, total − pipiens)) ensures that the number of individuals per trap is explicitly accounted for in the models. Models were fitted using maximum likelihood. Model performance was compared using Akaike’s Information Criterion corrected for small sample size (AICc). For each landscape–response combination, the buffer radius minimising AICc was considered the most supported scale of effect. Radii with ΔAICc < 4 were considered to have comparable support. To derive a single representative scale per landscape variable, we combined consistency across response variables with model support. For agricultural cover, the most frequently selected optimal radius, which was also within the ΔAICc < 4 confidence set for all models, was retained. For urban and forest cover, where the most frequently selected scale was supported in only a subset of models, we selected the radius that fell within the ΔAICc < 4 confidence set for the largest number of response variables. This approach ensured comparability among predictors while avoiding overparameterisation. Models using the fully optimised spatial scale for each landscape–response variable combination were also fitted, and the corresponding results are provided in the Supplementary Materials (Fig. S2).

### Environmental determinants of ecotype presence and relative frequency

Following the identification of optimal spatial scales, we fitted multivariate GAMs to assess the combined effects of landscape composition, meteorological conditions, date and sampling site on *C. pipiens* ecotype presence and relative frequency. Models included standardised landscape predictors at their selected spatial scale, as well as standardised temperature and rainfall variables. Sampling site was included as a random-effect term, and capture date was modelled as a smooth temporal function. Binomial error distributions were used in all models. Models were fitted using restricted maximum likelihood. To quantify uncertainty around parameter estimates, we computed 95% confidence intervals using a bootstrap procedure (1,000 resamples at the trap level). Statistical significance was also assessed using Wald tests. Model diagnostics indicated appropriate model fit across all GAMs. All models converged without numerical issues, and no evidence of insufficient basis dimension was detected for the temporal smooth (k-index values ranged from 0.93 to 1.12, all *P* > 0.25).

## Results

Overall, 3,326 mosquitoes were collected across 57 sampling sites, including 2,939 females and 387 males. Based on morphological identification of females, 1,878 individuals belonged to *C. pipiens/torrentium*, 18 to *Culex hortensis*, 16 to *Culex* sp., 23 to *Aedes albopictus*, 135 to *Aedes cinereus*, 247 to *Aedes* sp., 497 to the *Anopheles claviger* complex/*Anopheles plumbeus*, 4 to *Anopheles* sp., 96 to *Coquillettidia richiardii*, and 25 to *Culiseta* sp. The complete distribution of species across sampling sites is provided in the Supplementary Materials (Table S1).

Among the *C. pipiens/torrentium* samples, 1,566 individuals were molecularly identified as *C. pipiens*, one as *C. torrentium*, and 311 remained undetermined due to failed amplification or ambiguous bands on agarose gels. Within the identified *C. pipiens*, 1,018 were assigned to *C. p. pipiens*, 288 to *C. p. molestus*, 180 were classified as hybrids, and 80 remained undetermined at the ecotype level due to ambiguous molecular results. Ecotype distribution varied markedly across the study area, with some sites dominated by a single form and others exhibiting mixed assemblages (Fig. 1C, Table S2).

**Fig. 2:**
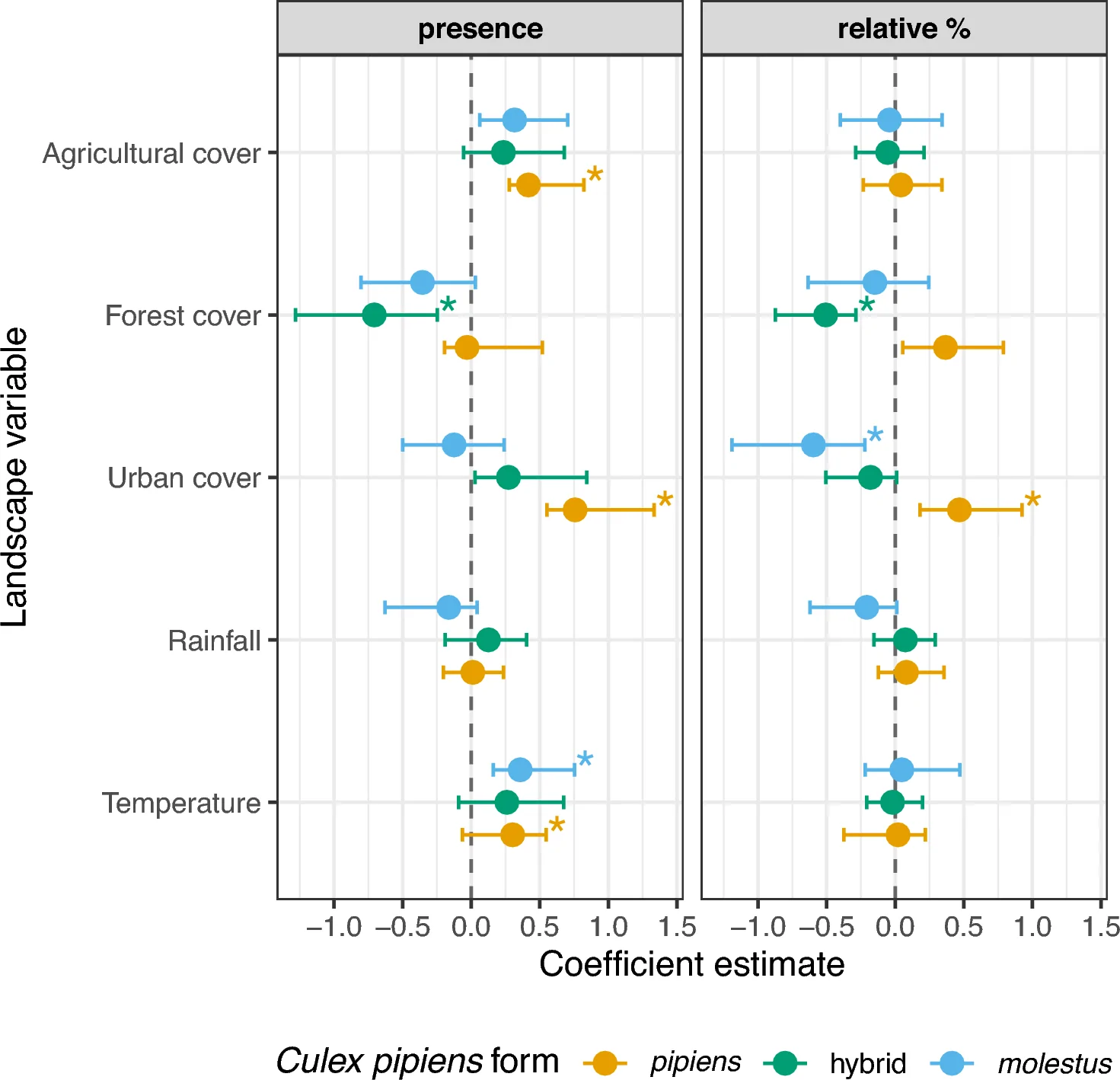
Estimated effects of landscape and meteorological variables on the presence and relative proportion of *C. pipiens* ecotypes (*pipiens*, *molestus*, hybrid). Points represent standardised model coefficients from generalised additive models, and horizontal bars show 95% confidence intervals calculated using a bootstrap procedure (1,000 resamples at the trap level). Stars indicate coefficients that are statistically significant based on Wald tests

No marked differences were observed among *C. pipiens* ecotypes in the spatial scale of effect of the three landscape variables (Fig. S3). However, urbanisation consistently operated at a broader spatial scale than forest or agricultural habitats. Based on the scale-of-effect analysis, representative buffer radii of 200 m were retained for forest and agricultural cover, and 850 m for urban cover (Fig. S3). Effect sizes along these landscape gradients revealed three distinct patterns. In agricultural areas, mosquito presence increased for all *C. pipiens* ecotypes, resulting in no marked change in their relative frequencies along the gradient (Fig. 2). In forested areas, hybrids decreased whereas *C. p. pipiens* and *C. p. molestus* remained unaffected, leading to a reduced proportion of hybrids with increasing forest cover (Fig. 2). Urbanisation positively affected *C. p. pipiens* but not *C. p. molestus* or hybrids. Accordingly, the proportion of *C. p. pipiens* increased and that of *C. p. molestus* decreased in urban areas, while no clear change in the proportion of hybrids was observed (Fig. 2).

Regarding meteorological variables, daily mean rainfall had no significant effect on *C. pipiens* ecotype presence and relative frequency. Daily mean temperature showed an overall positive effect on all *C. pipiens* ecotype presence, resulting in relatively stable proportions (Fig. 2).

**Fig. 3:**
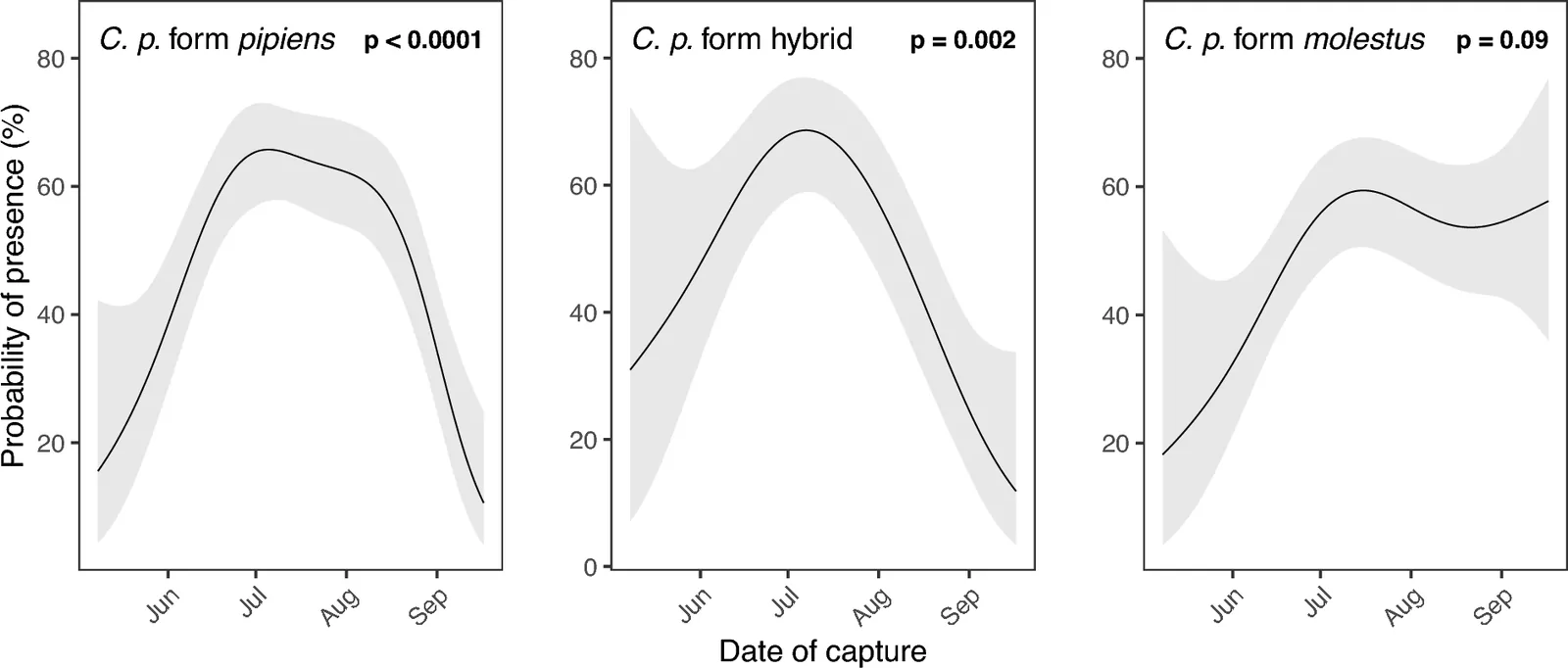
Temporal dynamics of the probability of presence of each *C. pipiens* ecotype over the 2025 capture season. Black lines show smooth trends fitted using generalised additive models, with shaded grey bands representing 95% confidence intervals

Seasonal variation in capture probability showed an increase at the beginning of the season for all *C. pipiens* ecotypes (Fig. 3). However, capture probability declined towards the end of the season for *C. p. pipiens* and hybrids, while no clear decrease was observed for *C. p. molestus*. The seasonal effect was significant for *C. p. pipiens* (smooth term: *χ*^2^ = 26.45, edf = 3.8, *P* < 0.0001) and hybrids (smooth term: *χ*^2^ = 15.84, edf = 3.4, *P* = 0.002), but not for *C. p. molestus* (smooth term: *χ*^2^ = 6.29, edf = 2.7, *P* = 0.09). Sampling site had a significant effect on ecotype presence for all three ecotypes, and on relative frequencies for *C. p. pipiens* and hybrids (*P* < 0.0001), indicating residual spatial variation not fully explained by the environmental predictors.

## Discussion

While *C. p. pipiens* showed a clear positive association with urbanisation, the absence of this association for *C. p. molestus* contrasts with the traditional view of this ecotype as the “urban” ecotype. This pattern is also reflected in the composition of co-occurring ecotypes, with the increase in *C. p. pipiens* likely contributing to the lower proportion of *C. p. molestus* in urban areas. At least in our study region, urban environments may offer a range of suitable larval habitats and host resources for *C. p. pipiens*, including abundant passerine bird communities [16, 45] and anthropogenic water containers [46]. Previous studies report contrasting patterns regarding the association between *C. pipiens* ecotypes and urban environments. Some found higher proportions of *C. p. pipiens* in urban areas [19, 20], or reported no clear urban effect for *C. p. molestus* [16, 20]. Conversely, increased frequencies of *C. p. molestus* in urban habitats have also been documented [17, 47], and *C. p. pipiens* has been associated with more natural or rural environments in certain regions [16, 18]. Together, these findings highlight the absence of a consistent urban–natural segregation pattern across Europe and indicate that the ecological distribution of *C. pipiens* ecotypes cannot be generalised but should instead be evaluated at the regional scale [19]. Importantly, our sampling was conducted exclusively outdoors and above ground. As *C. p. molestus* is often associated with indoor and belowground habitats [17, 48, 49], our approach may underestimate its occurrence in urban environments. Complementary sampling targeting indoor and belowground sites would be necessary to assess whether such micro-habitats influence the landscape patterns reported here.

Estimated effects of agricultural cover on occurrence were consistently positive across *C. pipiens* ecotypes, with largely overlapping confidence intervals, resulting in no detectable change in relative frequencies along this gradient. This pattern is consistent with several European studies reporting high abundances and frequent co-occurrence of *C. pipiens* ecotypes in arable and heterogeneous agricultural areas [50, 51]. Agricultural landscapes may provide a combination of open breeding sites, irrigation systems, and abundant avian hosts, creating favourable conditions for all *C. pipiens* ecotypes. Such environments have also been described as potential hybridisation zones, particularly in non-irrigated arable land where hybrids can occur at high frequencies [51]. The concordance between our findings and these studies suggests that agricultural habitats may represent important ecological interfaces facilitating contact between *C. pipiens* ecotypes.

In forested areas, hybrids showed a larger decline compared to both *C. p. pipiens* and *C. p. molestus*. This was the only landscape context in which hybrids did not display an intermediate response between *C. p. pipiens* and *C. p. molestus*. Hybridisation within the *C. pipiens* complex is thought to depend primarily on spatial overlap and contact between ecotypes, which can vary across habitat types. While agricultural and peri-urban environments have frequently been described as zones of co-occurrence and increased hybridisation [18, 51], we found no clear evidence in the literature linking forest habitats specifically to hybrid presence or proportion. Forested environments may reduce encounter rates between *C. p. pipiens* and *C. p. molestus* by promoting distinct microhabitat use or host associations, thereby limiting opportunities for interbreeding. Alternatively, forests may exert ecological filtering if hybrids display reduced fitness under more natural conditions. However, all these hypotheses remain untested and would require targeted ecological and experimental investigations.

In our study, temperature showed an overall positive association with the presence of all *C. pipiens* ecotypes. We expected *C. p. molestus* to exhibit greater thermal sensitivity because it is typically associated with warm, stable, confined habitats and may be less adapted to cooler environments, potentially limiting its occurrence above ground in northern European regions [9, 21]. However, this pattern was not clearly supported by our results. Several field studies have linked higher mean temperatures to increased *C. pipiens* abundance [52, 53], and elevated temperatures are known to accelerate larval development and shorten generation times in *Culex* species [54]. Together, these findings provide a plausible explanation for the positive relationship between temperature and ecotype presence detected here.

In contrast, rainfall did not show any effect on ecotype presence. Rainfall has often been positively associated with the abundance of *C. pipiens* ([52, 55], but see [56]) and other mosquito species [53], likely through its role in generating or maintaining suitable larval habitats. Our study area is located in a temperate region, where rainfall is relatively frequent and less limiting for mosquito breeding compared to regions with more variable or scarce precipitation, which may explain the absence of a detectable rainfall effect. However, this result may also reflect a mismatch between mosquito responses and the spatial and temporal resolution of our meteorological data. Mosquito oviposition and larval success may respond strongly to short-term rainfall events and fine-scale variation in local water availability (e.g. small containers, puddles, or ditches), which may not be captured by daily averages.

Seasonal dynamics differed markedly between the two *C. pipiens* ecotypes. Over the course of the summer, *C. p. pipiens* exhibited a marked peak in July followed by a sharp decline, whereas the probability of presence of *C. p. molestus* showed no clear change between July and late September. During colder periods before (April) or after (November) the main activity season of *C. p. pipiens*, *C. p. molestus* can still be captured and often represents the only ecotype present in Europe [39] or Asia [40]. This difference in seasonal trajectories reflects the inability of *C. p. molestus* to enter diapause, in contrast to *C. p. pipiens* [9]. Indeed, diapause cannot be induced in *C. p. molestus* even under experimental manipulation of photoperiod and temperature [41, 42]. These results highlight the persistence of *C. p. molestus* late in the season and given the more anthropophilic tendencies of this ecotype [10, 11], such seasonal shifts in biotype composition may have important implications for late-season arbovirus transmission dynamics [43], particularly under climate change [44]. As not all sites were sampled consistently throughout the season, our sampling design does not allow detailed characterisation of fine-scale seasonal dynamics at the level of individual sites. More consistent temporal monitoring within individual sites would help disentangle local seasonal trajectories and complement the overall seasonal trends across the study area reported here.

Hybrid individuals in our study largely followed the seasonal trajectory of *C. p. pipiens* that may reflect shared diapause characteristics. However, Fonseca et al. [12] suggested that the relatively low hybridisation rates in northern Europe are partly due to the limited ability of hybrids to enter diapause. Most populations from southern Europe are described as *C. p. pipiens*-like or exhibiting intermediate traits between the two biotypes, reflecting a continuum rather than discrete forms [9]. In this context, the seasonal trajectory observed for hybrids in our study may reflect the retention of functional diapause traits, possibly resulting from asymmetric introgression driven by the higher local abundance of *C. p. pipiens*. This hypothesis remains to be formally tested. Hybridisation rates in our study (11%) are consistent with patterns reported across Europe, where frequencies are typically low in northern regions (e.g. 1–5% in England [20] and Sweden [19]), intermediate in temperate areas (around 6–20% in Austria [51], the Netherlands [19], Italy [15, 19] or Portugal [18]), and can reach higher values in southern regions or Mediterranean contexts (up to 30–40% in Tunisia [17] or southern Spain [16]). This latitudinal and ecological variability likely reflects differences in environmental conditions and opportunities for contact between ecotypes.

Finally, the significant effect of sampling site suggests that unmeasured local factors may also contribute to shaping *C. pipiens* ecotype distribution, including microhabitat characteristics or fine-scale environmental conditions not captured by the landscape or meteorological variables used in this study. In addition, whether locally dominant *C. p. molestus* populations observed at some rural sites reflect stable overwintering populations or transient seasonal occurrences remains unknown and would require multi-year monitoring and targeted investigation of overwintering habitats. We also acknowledge that the CQ11 marker used in this study provides an operational rather than fully genomic definition of *C. pipiens* ecotypes. In regions where hybridisation and introgression are common, this marker may not fully capture complex patterns of ancestry, including backcrossing events that can lead to mismatches between genetic assignment and ecological or behavioural traits [9]. As a result, the ecological groups defined here should be interpreted as functional ecotypes relevant for vector ecology and surveillance rather than strictly genetically distinct lineages. Multi-locus or genomic approaches would be required to fully resolve patterns of introgression within the *C. pipiens* complex.

## Conclusions

Landscape effects on *C. pipiens* ecotypes are complex and vary depending on both the ecotype and the landscape variable considered. This highlights the need for extensive field-based studies across diverse regions to determine whether broader patterns exist or if *C. pipiens* ecotype distributions are highly specific to each area due to local adaptations or environmental conditions. Understanding these patterns is particularly important given the ongoing and projected expansion of arboviruses under global climate change in Europe. Future research should focus on virus surveillance, particularly for Usutu and West Nile viruses, and on landscape genetics to assess ecotype connectivity, the influence of landscape structure on gene flow, and thus identify important genetic differences between *C. pipiens* ecotypes.

## Supplementary Information

Additional file 1.

## Data Availability

Raw data and the R script supporting the findings of this study are publicly available in Mendeley Data (https://doi.org/10.17632/9kz55nnjky.1).
