# Supplementary material for: Divergent responses to urbanisation and forest cover shape the spatial distribution of Culex pipiens ecotypes in western Switzerland
Source: Parasit Vectors. 2026 Jun 26;19:395. doi: 10.1186/s13071-026-07533-8 (PMC13573415; doi:10.1186/s13071-026-07533-8)
Supplement: Supplementary file 1 [file 13071_2026_7533_MOESM1_ESM.pdf]

Table S1: Number of female mosquitoes by species at each of the 57 sampling sites.

Site	Latitude	Longitude	Mosquito species									
			Culex pipiens /torrentium	*Culex hortensis*	*Culex sp.*	*Aedes albopictus*	*Aedes cinereus*	*Aedes sp.*	*Anopheles claviger complex/Anopheles plumbeus*	*Anopheles sp.*	*Coquilletidia richiardii*	*Culiseta sp.*
ABB	46.66075	6.32781	2		1				1			
AIG	46.31913	6.97013	1						1			
AL	46.93528	7.02913	33					50	6		17	
BBA	46.85281	6.69356	54		3				2		3	
BCH	46.88087	6.91922	21		1			2	16		2	2
BE	46.5712	6.69599	21					2	3		2	
BGR	46.68916	6.79968	51						3			
BIE	46.53694	6.33247	21									3
BO	46.6525	6.56062	49					4	14	1	4	1
BRA	46.59704	6.22808	10				77	2	1	1	2	2
BRF	46.55189	6.15924	2									
BRO	46.62804	6.7573	43		1				57			
BU	46.68749	6.61608	41		1		1		1		1	
CBV	46.51938	6.60137				13						
CHA	46.46565	6.41052	23				1	3	15			
CHE	46.43966	6.23476	2	1				5	28		1	
COM	46.46276	6.28501							19			
COP	46.31682	6.19099	2									
COS	46.61406	6.50703	105						1			
CR	46.79142	6.69936	7				18	49	13	1	20	1
DES	46.68751	6.71465	33					3	25			
DEV	46.48678	6.89665	32		2			2	26			
DNS	46.51835	6.45765	11						1			
DO	46.52397	6.57843	42	11	3			4	48		1	
ECF	46.55025	6.47395	41						4			3
ECH	46.64086	6.63469	28									1
ECI	46.53006	6.50045	34		1				6			1
ERB	46.56697	6.84324	31						12			
ET	46.78152	6.55247	65						2			
FA	46.72504	6.91516	53				1	4	10		12	
FE	46.57369	6.392	8					39	6			5
FSA	46.53435	6.63979						1	1			
GIN	46.41057	6.17835	3	1								
GLA	46.4208	6.27129	38	1					1			
GO	46.56574	6.53215	22				3	9	33	1	1	
ISL	46.62041	6.41373	2									
LAU	46.51319	6.62255	349	1				1				
LUT	46.50855	6.68274	4					1				
MO	46.66112	6.45576	38					2	12			
MON	46.7787	6.75181	1		1			1	6			
MOU	46.66875	6.79789	33						2			
NYO	46.38337	6.23533	49			6			2			
OR	46.61106	6.60137	37	1			8	22	15			1
ORB	46.72891	6.53256	29	1				1	3		1	
PAY	46.82217	6.94226	39						4			
RAP	46.82286	6.60901							10			
RGR	46.39079	6.91043	39		1		25	23	10		27	
RI	46.49165	6.47869	34				1	9	14			1
RO	46.52492	6.29356	25		1				9			
ROB	46.5253	6.37042	21						3			
ROL	46.46129	6.34006	22					1	5			
SAV	46.53818	6.73161	60					2				1
TE	46.50592	6.80924	28					3	41			
VAL	46.7128	6.37908	1						1			1
VEV	46.46235	6.84413	22			4		1	3		1	2
VIL	46.39223	6.92846	48						1			
YVE	46.77391	6.64222	68	1				1			1	

Table S2: Number of each *C. pipiens* ecotypes (*pipiens, molestus*, hybrid) in the 57 sampling sites.

Site	*C. p. pipiens*	*C. p. molestus*	*C. p. hybrid*
ABB	1	1	
AIG	1		
AL	8	2	2
BBA	5	4	3
BCH	5	2	
BE	8	4	1
BGR	9	4	4
BIE	1	3	2
BO	7	3	2
BRA	5	1	1
BRF	2		
BRO	5	11	2
BU	8	7	3
CBV			
CHA	9	4	3
CHE	2		
COM			
COP	2		
COS	5	5	4
CR	3	1	1
DES	1	6	2
DEV	9	4	2
DNS	3	2	1
DO	12		1
ECF	9	8	4
ECH	7	3	4
ECI	5	9	4
ERB	11	5	2
ET	6	2	3
FA	14	4	2
FE	2	3	1
FSA			
GIN		1	1
GLA	4	2	3
GO	12	2	
ISL		1	1
LAU	5	2	5
LUT	2		
MO	1	6	2
MON		1	
MOU	7	2	3
NYO	8	5	7
OR	14	4	3
ORB	4	2	2
PAY	5	2	3
RAP			
RGR	9	2	2
RI	12	4	5
RO	8	5	1
ROB	2	9	4
ROL	9	1	2
SAV	4	4	2
TE	6	2	2
VAL			
VEV	7	4	
VIL	3	4	4
YVE	5	1	4

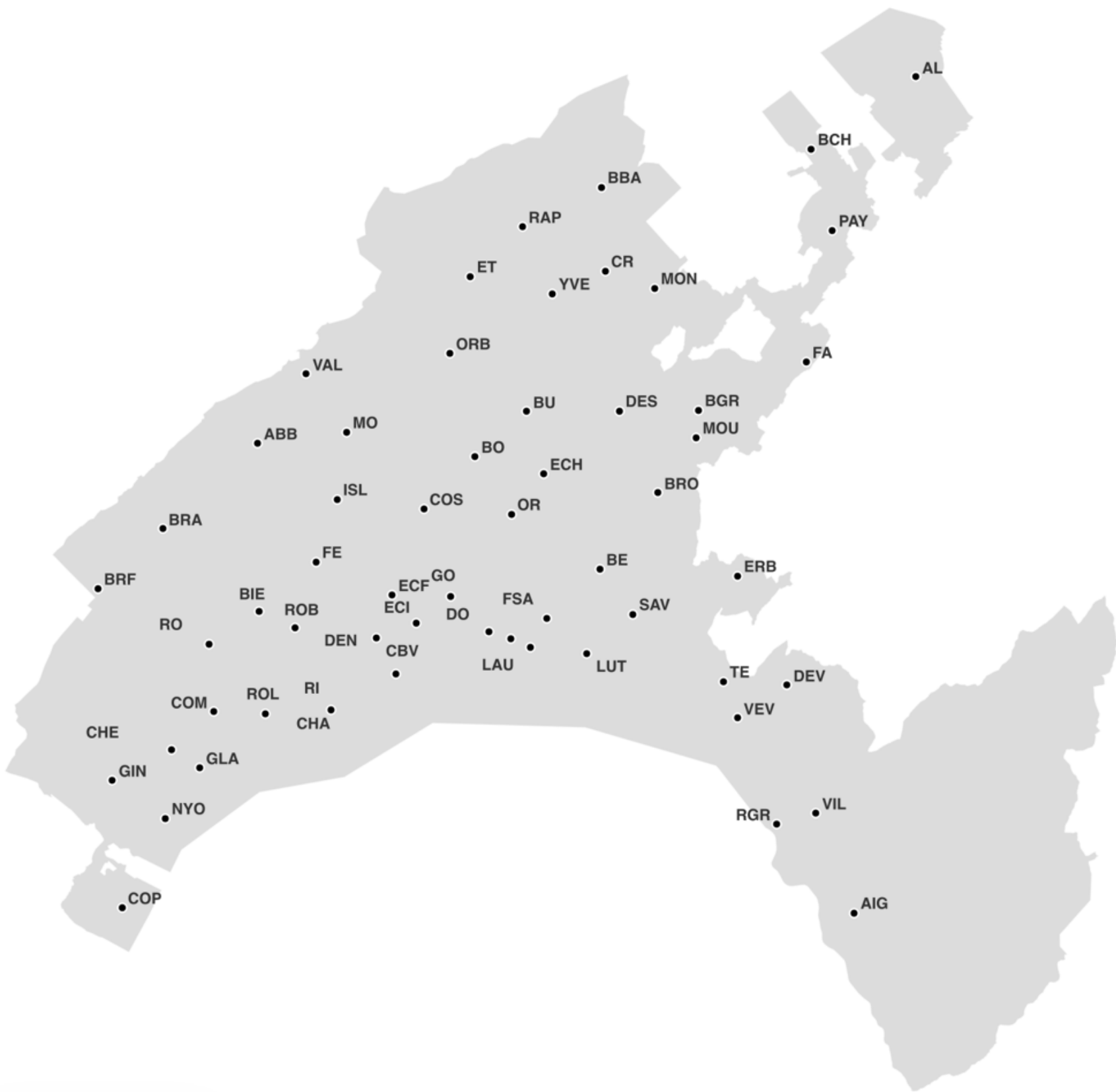

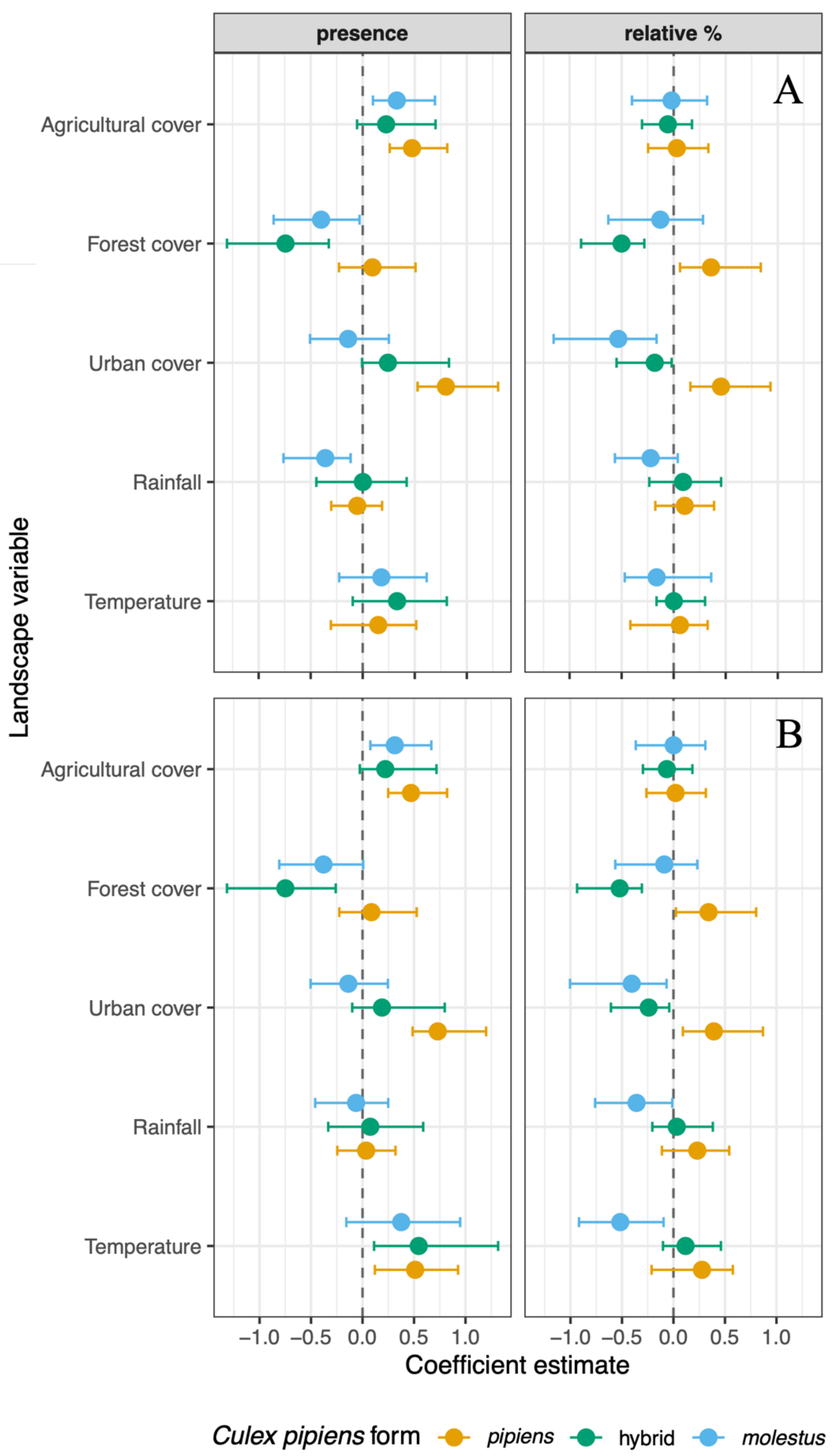

Fig. S1: Estimated effects of landscape and meteorological variables on the presence and relative proportion of *C. pipiens* ecotypes (*pipiens*, *molestus*, hybrid). Points represent standardised model coefficients from generalised additive models, and horizontal bars show 95% confidence intervals calculated using a bootstrap procedure (1,000 resamples at the trap level). The effects of rainfall and temperature were calculated as the mean values over (A) the seven days and (B) the 14 days preceding sampling.

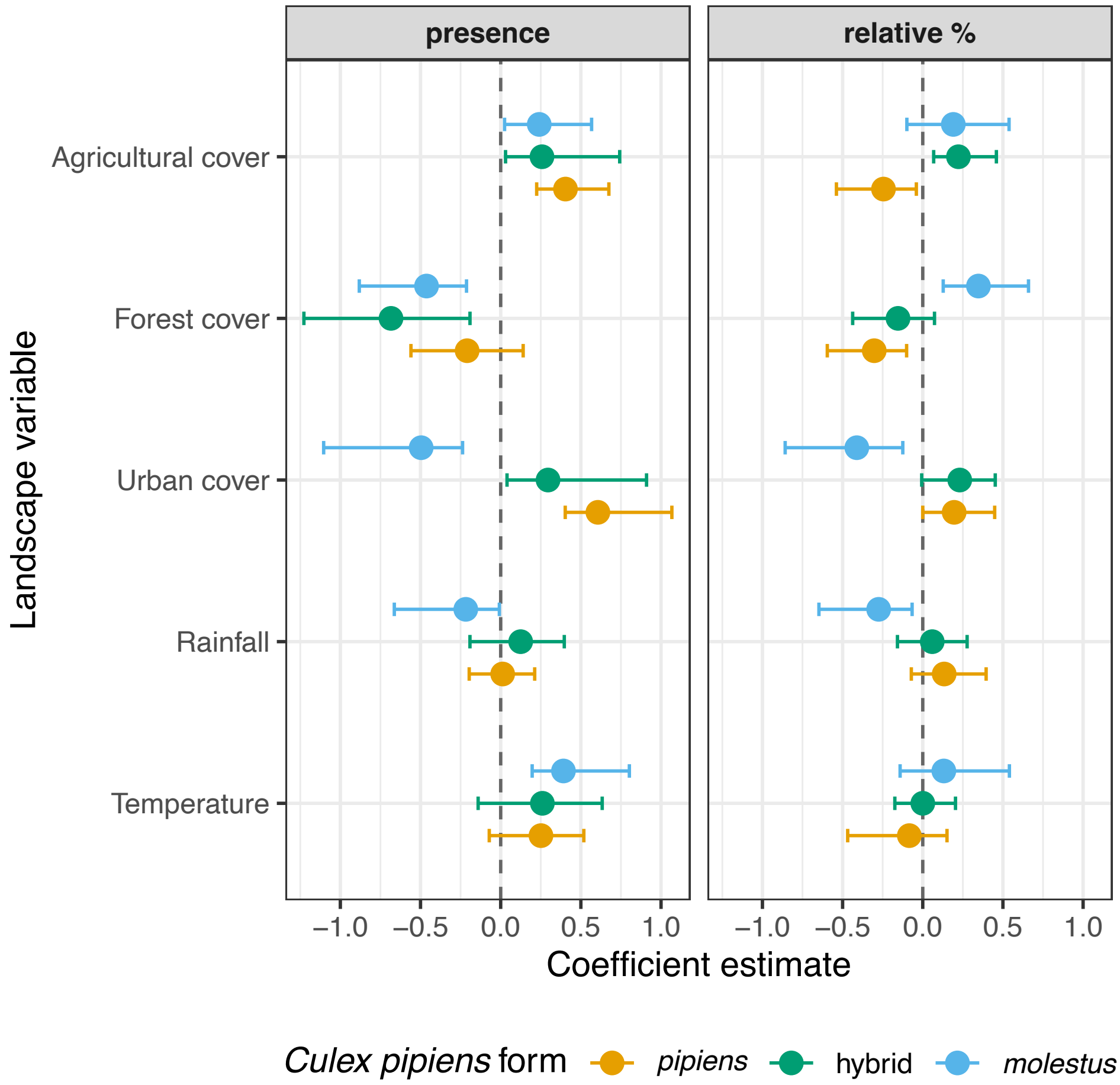

Fig. S2: Estimated effects of landscape and meteorological variables on the presence and relative proportion of *C. pipiens* ecotypes (*pipiens*, *molestus*, hybrid). Points represent standardised model coefficients from generalised additive models, and horizontal bars show 95% confidence intervals calculated using a bootstrap procedure (1,000 resamples at the trap level). Landscape effects were estimated at the spatial scale with the lower AICc for each landscape-response variable combination.

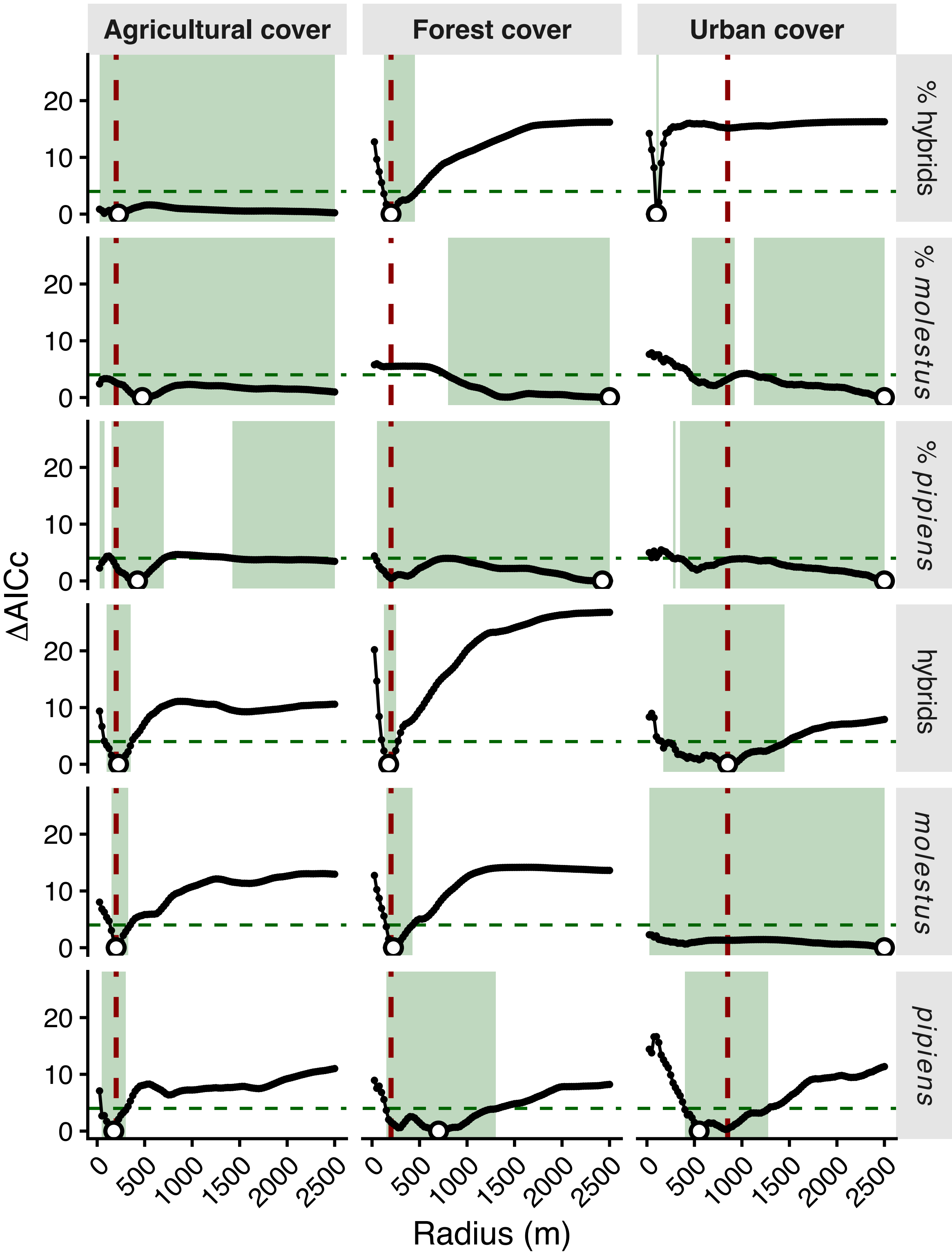

Fig. S3: Scale-of-effect of agricultural, forest, and urban cover on the presence and relative abundance of *C. pipiens* ecotypes. ΔAICc values compare each model to the best-fitting model (white points) across buffer radii for each landscape-response combination. Shaded green bands indicate radii with ΔAICc ≤ 4, considered equally supported as the best spatial scale. Horizontal dashed green lines show the ΔAICc = 4 threshold. The vertical dashed red line indicates the buffer radius selected for the full effect-size analyses. Names without % indicate presence (e.g., *pipiens*), while labels with % indicate the relative proportion of each ecotype (e.g., % *pipiens*).
